# Cerebrospinal Fluid Proteomics in Friedreich Ataxia Reveals Markers of Neurodegeneration and Neuroinflammation

**DOI:** 10.3389/fnins.2022.885313

**Published:** 2022-07-13

**Authors:** Virginie Imbault, Chiara Dionisi, Gilles Naeije, David Communi, Massimo Pandolfo

**Affiliations:** ^1^Mass Spectrometry and Proteomics Laboratory/Platform, Institut de Recherche en Biologie Humaine et Moléculaire (IRIBHM), Université Libre de Bruxelles, Brussels, Belgium; ^2^Laboratory of Experimental Neurology, Université Libre de Bruxelles, Brussels, Belgium; ^3^Neurology Service, Hôpital Erasme, Université Libre de Bruxelles, Brussels, Belgium

**Keywords:** Friedreich Ataxia, cerebrospinal fluid, proteomics, neuroinflammation, biomarker, neurodegeneration

## Abstract

Clinical trials in rare diseases as Friedreich ataxia (FRDA) offer special challenges, particularly when multiple treatments become ready for clinical testing. Regulatory health authorities have developed specific pathways for “orphan” drugs allowing the use of a validated biomarker for initial approval. This study aimed to identify changes in cerebrospinal fluid (CSF) proteins occurring in FRDA patients that may be potential biomarkers in therapeutic trials. CSF was obtained from 5 FRDA patients (4 females, 1 male) from the Brussels site of the European Friedreich Ataxia Consortium for Translational Studies (EFACTS). Two patients were ambulatory, three used a wheelchair. Residual CSF samples from 19 patients who had had a lumbar puncture as part of a diagnostic workup were used as controls. All CSF samples had normal cells, total protein and glucose levels. Proteins were identified by label-free data-dependent acquisition mass spectrometry (MS) coupled to micro-high performance liquid chromatography. We found 172 differentially expressed proteins (DEPs) (92 up, 80 down) between FRDA patients and controls at *P* < 0.05, 34 DEPs (28 up, 6 down) at *P* < 0.0001. Remarkably, there was no overlap between FRDA patients and controls for seven upregulated and six downregulated DEPs. Represented pathways included extracellular matrix organization, signaling, the complement cascade, adhesion molecules, synaptic proteins, neurexins and neuroligins. This study supports the hypothesis that the quantitative analysis CSF proteins may provide robust biomarkers for clinical trials as well as shed light on pathogenic mechanisms. Interestingly, DEPs in FA patients CSF point to neurodegeneration and neuroinflammation processes that may respond to treatment.

## Introduction

Friedreich ataxia (FRDA) is characterized by a specific pattern of clinical features and neuropathology, evolving in time (Harding et al., 2020). Afferent ataxia, due to altered development and loss of proprioceptive sensory neurons in the dorsal root ganglia (DRGs), appears first. Cerebellar ataxia, due to atrophy of the dentate nucleus, leads to progression of symptoms. Degeneration of the pyramidal tracts becomes prominent in advanced disease, causing weakness and spasticity.

In almost all cases, the underlying genetic mutation is the hyperexpansion of a GAA repeat in the first intron of the *FXN* gene, encoding frataxin (Campuzano et al., 1996). The expanded repeats suppress *FXN* expression by triggering chromatin condensation (Yandim et al., 2013). Frataxin is a component of the protein complex that synthesizes iron-sulfur (Fe-S) clusters in the mitochondrial matrix (Fox et al., 2015). Its deficiency leads to multiple metabolic abnormalities due to loss of Fe-S proteins activities, altered iron homeostasis and oxidative stress (Synofzik et al., 2019). However, the specific pathogenic mechanisms leading to the characteristic FRDA neuropathology have not yet been discovered.

As new therapeutic strategies aiming to restore frataxin levels or to correct the consequences of its deficiency move to clinical development, trial design becomes a critical issue. FRDA is a rare disease, so there is a limited number of patients that can participate in trials. Furthermore, its relatively slow progression requires trials of relatively long duration (18–24 months) to reliably detect clinical changes (Reetz et al., 2021). Biomarkers may help circumventing these issues by allowing to initially evaluate a new treatment in a smaller cohort and a shorter time, providing guidance about the continuation of the trial.

There is a need for biomarkers reflecting central nervous system (CNS) pathology and response to treatment in FRDA. Neuroimaging (Harding et al., 2021) and neurophysiology (Naeije et al., 2019) biomarkers show promise, but there are few data about changes in the cerebrospinal fluid (CSF). The CSF contains low levels of a variety of proteins, some of which are affected by pathological changes. We performed a pilot study of CSF proteomics in FRDA to detect differentially expressed proteins (DEPs) in FRDA patients compared to control individuals. We hypothesized that these DEPs may be involved in disease pathogenesis and their levels may be corrected by therapies.

The main previous finding about biofluid biomarkers in FRDA is the increased level of neurofilament light chain (NfL) in blood (Clay et al., 2020). Neurofilament light chain is a major cytoskeletal protein with a role in axon growth and stability and in synaptic organization. Its level is increased in biofluids of patients with neurological disorders, but the mechanisms of their release from neurons and their relationship with neurodegenerative and neuroregenerative processes are still poorly understood (Gafson et al., 2020). The use of NfL as biomarker in FRDA patients is restricted to those younger than 25 years old, as blood NfL levels after this age overlap in FRDA patients and controls (Clay et al., 2020). Overall, there is an unmet need for reliable biofluid biomarkers in FRDA.

Recent progresses in data acquisition and analysis with mass spectrometry have led to development of the Sequential Window Acquisition of all Theoretical fragment-ion spectra (SWATH-MS) method (Gillet et al., 2012). This approach offers a better proteomic coverage (up to 1000–1500 proteins with <200 μl of analyzed cerebrospinal fluid) as well as more reproducible identification and quantification of thousands of proteins from complex protein mixtures with performance characteristics that approach those of multiple/selected reaction monitoring. Moreover, once acquired, the data can be perpetually analyzed *in silico* to test new potential biomarkers (Liu et al., 2014). Our unbiased proteomic study on the CSF of five FRDA patients revealed significant changes in proteins belonging to several pathways involved in neurodegeneration and neuroinflammation. For some proteins, levels did not overlap with those in control CSF samples, suggesting that they may become promising biomarkers for clinical trials.

## Materials and Methods

Cerebrospinal fluid (CSF) was obtained by lumbar puncture from 5 FRDA patients (4 females, 1 male) after written informed consent. These patients were enrolled in the European Friedreich Ataxia Consortium for Translational Studies (EFACTS) clinical study, for which they had previously provided written informed consent. All study protocols and materials for informed consent had been approved by the Erasme Hospital Ethics Committee.

After collection, an aliquot of CSF was sent to the Erasme Hospital diagnostic laboratory for cell count, protein and glucose determination, the rest was centrifuged, frozen, and stored in low protein-binding vials at –80°C until analysis.

As controls (CT), we used residual CSF samples form 19 patients (12 females and 7 males, median age 33, range 20–62) who had had a lumbar puncture as part of a diagnostic workup. Residual biological material was used as established by the Erasme Hospital Ethics Committee. Final diagnoses included primary headache (15 patients), functional neurological disorder (3 patients), and idiopathic intracranial hypertension (1 patient). All had a normal neurological exam. All FRDA and CT CSF samples had normal cells (median 1, range 0–3 white blood cells/mm^3^), protein and glucose values according to the laboratory norms.

For analysis, CSF samples were normalized by volume. Total protein concentration was measured to ensure homogeneity of protein content across samples, no other quality control test was performed. Proteins were identified by label-free data-dependent acquisition (DDA) mass spectrometry (TripleTOF 5600 SWATH, AB Sciex) coupled to micro-high performance liquid chromatography (μLC 425, Eksigent). Sample were passed on multi-affinity spin cartridges (ProteoPrep, Sigma) to remove non-specific abundant proteins (serum albumin and immunoglobulin G) and increase the dynamic range of protein detection. The concentrated samples were reduced and alkylated before tryptic digestion. Digested peptides were purified by using optimized multi-step StageTips C18 (ThermoFischer Scientific) before injection in the LC/mass spectrometry system. After hydrophobic separation on a column (Sciex μLC, 150 mm, 2.7 μm) using a two-step acetonitrile gradient with formic acid, peptides were sprayed online in the mass spectrometer. The 20 to 50 most intense precursors with charge state 2 to 4 were selected for fragmentation and MS2 spectra were collected in the range 100–2000 m/z for 100 ms. We excluded all spectra below 150.

In a second analysis, each sample was re-analyzed for protein quantification using SWATH acquisition mass spectrometry on the same instrument. The chromatographic parameters were as described above for the first analysis. For SWATH data acquisition, the instrument was tuned to allow a quadrupole resolution of 25 Da m/z mass selection. Using an isolation of 26 Da m/z, a set of 32 overlapping windows was constructed covering the precursor mass range of 400–1200 m/z. SWATH spectra were acquired from 100 to 2000 m/z.

The combined data were searched using ProteinPilot 4.5 (Sciex) using the Paragon algorithm (Sciex). The data were searched against the SwissProt database (Oct 2019). Decoy database search was performed with target false discovery rate <1% (FDR). Statistical analysis was performed using R language software v.3.2.1 and the statistical tools of the Protein Pilot, Paragorn and Peak Analyst^^®^^ software packages available with the TripleTOF 5600 SWATH mass spectrometer (AB Sciex). Data were represented as Log_2_(Mean Peak Intensity). We determined the profile of differentially expressed proteins (DEPs) between FRDA and CT individuals with a | Log_2_(Fold Change)| > 1 at *P* < 0.05 and *P* < 0.0001 (*t*-test, no correction for multiple comparisons). DEPs ontology and involved pathways were analyzed using Panther and Reactome software.

## Results

Patient data are shown in Table 1. Two patients were ambulatory (SARA scores: 11 and 16), three used a wheelchair (SARA scores: 26.5, 27.5, 33).

**TABLE 1:** Friedreich ataxia (FRDA) patients’ genetic and clinical data.

GAA1	Age of onset	Disease duration	SARA	M/F
450	20	29	26.5	F
445	15	8	16	F
712	7	45	33	F
445	23	8	11	M
780	18	15	27.5	F

We could quantify 2,523 peptides from 564 proteins in CT and FRDA CSF samples.

**FIGURE 1:**
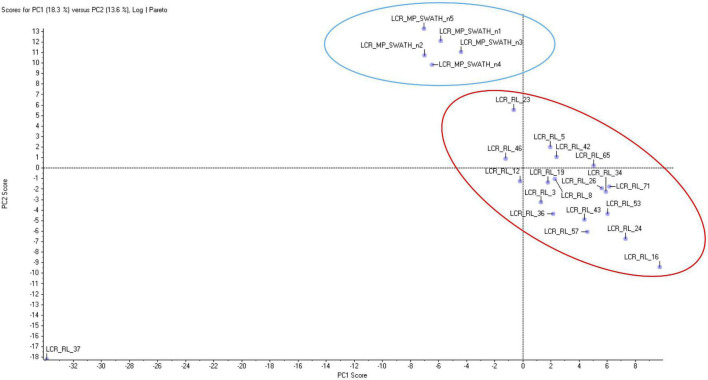
Principal component analysis of proteomic data from Friedreich ataxia (FRDA) and controls (CT) samples. The two groups form two homogeneous separated clusters.

Principal Component Analysis (PCA) revealed well defined and separated clusters for CT and FRDA samples (Figure 1), with a single CT outlier. Sample clustering indicated that results are reliable, with minimal random variability due to sample quality issues. We found 172 DEPs (92 up, 80 down) between FRDA patients and controls using an uncorrected *P* < 0.05 as cutoff. Among these, 34 DEPs (27 up, 6 down) were detected with uncorrected *P* < 0.0001 (Table 2 and Supplementary Material 1). Supplementary Material 2 include the number of unambiguously (confidence index > 95%) microsequenced peptides for each DEP of interest, as well as the related coverage percentage of the complete primary structure.

**TABLE 2:** Over- (red background) and under- (green background) represented differentially expressed proteins (DEPs) in Friedreich ataxia (FRDA) vs. control cerebrospinal fluid (CSF) at *P* < 0.0001.

Protein (gene name)	P value	Fold change	Log fold change
VPS10 domain-containing receptor SorCS3 (SORCS3)	1.33E-10	8.90	0.95
Transthyretin (TTR)	1.80E-06	5.56	0.75
Membrane protein FAM174A (FAM174A)	2.03E-10	4.48	0.65
Protein YIPF3 (YIPF3)	9.14E-05	3.53	0.55
Neuroendocrine protein 7B2 (SCG5)	1.16E-07	3.51	0.54
Isoform 2 of Neuroblastoma suppressor of tumorigenicity 1 (NBL1)	8.70E-08	3.31	0.52
Glutathione peroxidase (GPX3)	5.34E-08	3.18	0.50
Trypsin-1 (PRSS1)	8.38E-06	3.12	0.49
Protein shisa-6 (SHISA6)	5.61E-08	3.10	0.49
Neurexophilin-4 (NXPH4)	3.89E-05	3.01	0.48
Isoform M1 of Pyruvate kinase (PKM)	1.33E-10	2.78	0.44
Guanine deaminase (GDA)	5.86E-05	2.75	0.44
Ribonuclease pancreatic (RNASE1)	5.98E-06	2.71	0.43
CD99 antigen (CD99)	4.80E-05	2.71	0.43
Gelsolin (GSN)	5.14E-05	2.65	0.42
Neurosecretory protein VGF (VGF)	5.86E-05	2.61	0.42
UPF0606 protein KIAA1549L (KIAA1549L)	7.68E-05	2.57	0.41
Chromogranin-A (CHGA)	5.91E-06	2.51	0.40
Macrophage colony-stimulating factor 1 (CSF1)	8.98E-07	2.50	0.40
Proenkephalin-A (PENK)	3.58E-05	2.50	0.40
Phosphatidylethanolamine-binding protein 1 (PEBP1)	2.14E-05	2.35	0.37
Beta-2-microglobulin (B2M)	2.65E-06	2.33	0.37
Lysosomal Pro-X carboxypeptidase (PRCP)	1.21E-08	2.25	0.35
Isoform 4 of Mannan-binding lectin serine protease 1 (MASP1)	3.62E-05	2.06	0.31
Collagen alpha-2(I) chain (COL1A2)	1.86E-05	1.73	0.24
Ubiquitin-40S ribosomal protein S27a (RPS27A)	1.11E-05	1.72	0.23
Cystatin-C (CST3)	3.55E-05	1.72	0.23
**Protein (gene name)**	**P value**	**Fold change**	**Log fold change**
Beta-Ala-His dipeptidase (CNDP1)	1.30E-07	0.18	–0.74
Fibulin-1 (FBLN1)	1.55E-05	0.20	–0.69
Complement C3 (C3)	3.72E-05	0.26	–0.59
Monocyte differentiation antigen CD14 (CD14)	2.65E-05	0.31	–0.50
Immunoglobulin superfamily containing leucine-rich repeat protein (ISLR)	1.20E-05	0.41	–0.39
Transforming growth factor-beta-induced protein ig-h3 (TGFBI)	2.68E-05	0.48	–0.32

Of notice, there was no overlap between FRDA patients and controls for seven over-represented (SORCS3, PKM, FAM174A, PRCP, GPX3, NBL1, SCG5) and six under-represented DEPs (CNDP1, ISLR, CD14, C3, C9, NRXN2), suggesting that these may be potential biomarkers for clinical studies.

Among the above over-represented DEPs, SORCS3 and SCG5 have their highest levels in the brain and have been associated with neurodegeneration. SORCS3 is a type-I receptor transmembrane protein whose postulated functions include a role in amyloid precursor protein processing (Reitz, 2015). Genetic variants of SORCS3 are risk factors for Alzheimer disease (AD). SCG5 is a secreted chaperone protein that suppresses aggregation of neurodegeneration related proteins such as amyloid-β (Aβ)-derived peptides and tau in AD and α-synuclein in Parkinson disease (PD) (Helwig et al., 2013). The other DEPs are present in a variety of tissues and have metabolic (PKM in carbohydrate and FAM174A in lipid metabolism), developmental (NBL1, a bone morphogenetic protein antagonist), protease (PRCP), or antioxidant (GPX3) functions.

The above under-represented DEPs include CNDP1, a secreted metalloprotease highly expressed in the brain; ISLR, an extracellular protein predicted to be involved in cell adhesion; the synaptic adhesion protein NRXN2, whose genetic variants are linked to neuropsychiatric diseases (Kasem et al., 2017); and three inflammation related proteins, the complement factors C3 and C9 and the macrophage protein CD14, with a postulated role in neurodegenerative and neuroinflammatory diseases (Yanamadala and Friedlander, 2010; Propson et al., 2020).

Panther and Reactome analysis detected over- and under-represented pathways in FRDA CSF. Components of several signaling pathways were over-represented in FRDA, in particular insulin-like growth factor (IGF) regulation by IGF binding proteins (IGFBPs), and more generally protein phosphorylation, extracellular matrix (ECM) organization, immune response, and the complement cascade (Table 3). Under-represented DEPs Table 4 in part belonged to the same pathways, particularly IGF regulation, ECM organization, protein phosphorylation and the complement cascade, plus platelet activation, ERBB4 signaling and receptor-type protein phosphatases.

**TABLE 3:** *Reactome* pathway analysis of over-represented differentially expressed proteins (DEPs) in Friedreich ataxia (FRDA) cerebrospinal fluid (CSF).

Pathway name	Entities				Reactions	
	found	ratio	*P-value*	FDR*	found	ratio
Post-translational protein phosphorylation	14/109	0.007	1.82e-13	1.24e-10	1/1	7.63e-05
Regulation of Insulin-like Growth Factor (IGF) transport and uptake by Insulin-like Growth Factor Binding Proteins (IGPBPs)	14/127	0.009	1.38e-12	4.71e-10	1/14	0.001
Neutrophil degranulation	16/480	0.033	7.95e-07	1.80e-04	9/10	7.63e-04
Extracellular matrix (ECM) organization	11/330	0.022	4.72e-05	0.008	81/319	0.024
Innate Immune System	24/1,331	0.09	5.56e-05	0.008	107/701	0.053
Immune System	37/2,895	0.197	5.92e-04	0.058	179/1,647	0.126
Amyloid fiber formation	5/88	0.006	6.03e-04	0.058	7/33	0.003
Degradation of the ECM	6/148	0.01	9.97e-04	0.085	20/105	0.008
Non-integrin membrane-ECM interactions	4/61	0.004	0.001	0.089	5/22	0.002
Complement cascade	6/156	0.011	0.001	0.089	25/71	0.005
Syndecan interactions	3/29	0.002	0.001	0.092	2/15	0.001

Only entities with p < 0.001 are shown. *False Discovery Rate.

**TABLE 4:** *Reactome* pathway analysis of under-represented differentially expressed proteins (DEPs) in Friedreich ataxia (FRDA) cerebrospinal fluid (CSF).

Pathway name	Entities				Reactions	
	found	ratio	*P-value*	FDR*	found	ratio
Extracellular matrix organization	16/330	0.022	1.44e-09	5.08e-07	72/319	0.024
Regulation of lnsulin-like Growth Factor (IGF) transport and uptake by Insulin-like Growth Factor Binding Proteins (IGFBPs)	10/127	0.009	2.60e-08	4.58e-06	9/14	0.001
Insulin-like Growth Factor-2 mRNA Binding Proteins	5/13	8.83e-04	4.38e-08	4.6le-06	2/3	2.29e-04
Platelet degranulation	10/137	0.009	5.24e-08	4.6le-06	3/11	8.39e-04
Response to elevated platelet cytosolic Ca2+	10/144	0.01	8.28e-08	5.20e-06	3/14	0.001
Post-translational protein phosphorylation	9/109	0.007	8.98e-08	5.20e-06	1/1	7.63e-05
ECM proteoglycans	8/79	0.005	1.04e-07	5.20e-06	8/23	0.002
Terminal pathway of complement	4/8	5.44e-04	3.70e-07	l.63e-05	5/5	3.8le-04
Protein-protein interactions at synapses	7/93	0.006	4.69e-06	l.83e-04	17/33	0.003
Molecules associated with elastic fibers	5/38	0.003	8.13e-06	2.85e-04	6/10	7.63e-04
Elastic fiber formation	5/46	0.003	2.02e-05	6.47e-04	8/17	0.001
Integrin cell surface interactions	6/87	0.006	3.73e-05	0.001	9/55	0.004
Platelet activation, signaling and aggregation	10/296	0.02	4.65e-05	0.001	18/115	0.009
Nuclear signaling by ERBB4	4/47	0.003	3.57e-04	0.009	4/34	0.003
Receptor-type tyrosine-protein phosphatases	3/20	0.001	4.0le-04	0.009	5/6	4.58e-04
HDL remodeling	3/24	0.002	6.79e-04	0.Ql5	6/13	9.92e-04
Neurexins and neuroligins	4/60	0.004	8.85e-04	0.Ql8	11/19	0.001
Formation of Fibrin Clot (Clotting Cascade)	4/61	0.004	9.4le-04	0.018	12/61	0.005
Activation of C3 and C5	2/7	4.76e-04	0.001	0.021	3/3	2.29e-04
Plasma lipoprotein assembly	3/30	0.002	0.001	0.022	2/19	0.001

*Only entities with p < 0.001 are shown.*

## Discussion

Although preliminary, this study supports the hypothesis that the quantitative analysis CSF proteins may provide biomarkers for clinical trials in FRDA as well as shed light on pathogenic mechanisms. FRDA is a rare disease, CSF analysis is not part of the diagnostic workup for this condition, and LP is an invasive procedure. About 10% of the 53 patients enrolled in EFACTS at our site consented to this study, which met our expectations.

Over-represented DEPs may include genuinely upregulated proteins from CNS cells, proteins from cells present in the CNS in increased number, as well as proteins that are shed by degenerating cells affected by FRDA. Conversely, under-represented DEPs may be downregulated proteins, or proteins normally shed by cells that are being lost in FRDA and therefore in lower number than in controls, or proteins that are consumed by some pathological process. While our data do not allow to reliably point to the cause of differential expression for all DEPs in FRDA CSF, we can make some educated guesses. Changes in neuronal proteins, particularly in synaptic proteins, strongly suggest neurodegeneration as the underlying mechanism (Tumani et al., 2014). The same goes for proteins involved in growth factor and other intracellular signaling, protein aggregation and metabolic function.

Changes in proteins involved in the immune response, in particular in the complement cascade, support the role of neuroinflammation (Kulic and Unschuld, 2016) in FRDA. In this regard, out findings are in general agreement with previous proteomic and transcriptomic analyses of peripheral cells and mouse models. In these, immune system activation occurs in all tissues and is among the earliest pathways regulated in the *Fxn* knockdown model (Chandran et al., 2017), with similar changes in the chemokine signaling pathway (*Ccl2, 3, 4, 7; Cxcl1, 16; Prkcd; Stat3*) occurring in other mouse models. Neuroinflammation has also been documented in DRGs (Koeppen et al., 2016), where degenerating large sensory neurons show invasion by satellite cells and inflammation. Hypertrophic microglia are present in the dentate nucleus of FRDA patients (Koeppen et al., 2012), and neuroinflammation is evident in the cerebellum and brainstem of FRDA patients (Khan et al., 2022). These results implicate microglia-mediated neuroinflammatory mechanisms in FRDA pathophysiology, similar to the dysregulated functions of microglia in other neurodegenerative diseases (Hickman et al., 2018; Rosenblum and Kosman, 2022).

Neurodegeneration and neuroinflammation are processes that may respond to treatment, so at least some of these DEPs may turn out to be treatment response biomarkers in addition to diagnostic biomarkers.

The major limitation of this study is its small sample size, which makes it preliminary, and its cross-sectional design. Furthermore, results need validation with specific protein assays. Nevertheless, we found a set of DEPs that show large differences in levels between FRDA patients and controls, which may be promising biomarkers. Studying a larger cohort of FRDA patients, possibly in a prospective study, will be needed to correlate DEPs with clinical and genetic data, refining their potential use in clinical trials.

## Data Availability Statement

The original contributions presented in this study are included in the article/Supplementary Material, further inquiries can be directed to the corresponding author.

## Ethics Statement

The studies involving human participants were reviewed and approved by Comité d’éthique – Hôpital Erasme, Brussels, Belgium. The patients/participants provided their written informed consent to participate in this study.

## Author Contributions

MP and DC conceived and supervised the study. GN assessed patients and obtained samples. VI carried out the experiments. VI, MP, and DC analyzed the data. MP wrote the manuscript. All authors read and approved the final manuscript.

## Conflict of Interest

The authors declare that the research was conducted in the absence of any commercial or financial relationships that could be construed as a potential conflict of interest. The authors declare that this study received funding from Voyager Therapeutics. The funder was not involved in the study design, collection, analysis, interpretation of data, the writing of this article or the decision to submit it for publication.

## Publisher’s Note

All claims expressed in this article are solely those of the authors and do not necessarily represent those of their affiliated organizations, or those of the publisher, the editors and the reviewers. Any product that may be evaluated in this article, or claim that may be made by its manufacturer, is not guaranteed or endorsed by the publisher.
